# Effects of “Danzhi Decoction” on Chronic Pelvic Pain, Hemodynamics, and Proinflammatory Factors in the Murine Model of Sequelae of Pelvic Inflammatory Disease

**DOI:** 10.1155/2015/547251

**Published:** 2015-12-31

**Authors:** Xiaoling Bu, Yanxia Liu, Qiudan Lu, Zhe Jin

**Affiliations:** ^1^Beijing University of Traditional Chinese Medicine, North 3rd Ring Road No. 11 School Range, Chaoyang District, Beijing 100029, China; ^2^Department of Gynecology, Dongfang Hospital of Beijing University of Traditional Chinese Medicine, No. 6 Fangxingyuan 1 Qu, Fengtai District, Beijing 100078, China

## Abstract

*Objective.* To evaluate the effect of Danzhi decoction (DZD) on chronic pelvic pain (CPP), hemodynamics, and proinflammatory factors of sequelae of pelvic inflammatory diseases (SPID) in murine model.* Methods.* SPID mice were randomly treated with high-dose DZD, mid-dose DZD, low-dose DZD, aspirin, and vehicle for 3 estrous circles. The Mouse Grimace Scale (MGS) was performed to evaluate CPP; blood flows of the upper genital tract, pelvic wall, and mesentery were used to assess hemodynamics in SPID mice; expressions of vascular endothelial growth factor (VEGF), angiopoietin-2 (Ang-2), and osteopontin (OPN) were measured by Western blot and immunochemistry.* Results.* Treatment with dose-dependent DZD significantly decreased the MGS scores, accelerated blood flows of the pelvis, and reduced expressions of VEGF, Ang-2, and OPN in the upper genital tract.* Conclusions and Discussions.* DZD was effective in relieving CPP and improving hemodynamics of the pelvic blood-stasis microenvironment in SPID mice. There was a relationship between CPP and the pelvic blood-stasis microenvironment. Furthermore, DZD might play a positive role in the anti-inflammatory process.

## 1. Introduction

PID is most commonly a complication of sexually transmitted infections (STIs), involving any or all of the uterus, oviducts, and/or ovaries [1]. PID may cause sequelae (i.e., chronic pelvic pain, ectopic pregnancy, and infertility) from tubal scarring and adhesions [2], which is called SPID and was once called chronic pelvic inflammatory disease (CPID).

In theory of traditional Chinese medicine (TCM), the main pathogenesis of SPID is blood stasis in collaterals of the pelvis. It refers to abnormal hemodynamics, such as the circulation of blood that is not smooth, blood flow that is stagnant or forms stasis [3], or the decrease of blood flow velocity. Pathogenic factors, like cold and dampness, damage the uterus and uterine collaterals and then lead to qi obstruction that further results in blood stasis in collaterals in the pelvis [4]. Blood stasis increases microvascular distortion and/or microvascular obstruction, which will aggravate stagnation of blood as well [5]. As a pathogenic factor, it directly affects the delivery of beneficial nutrients and clear of detrimental molecules or waste products. Therefore, blood stasis usually causes organ dysfunction and induces a series of symptoms, mostly in connection with slow-progressing diseases or chronic diseases [6]. In SPID, blood stasis affects the peristalsis of fallopian tubes so that it leads to abnormal pregnancy or infertility; because of microvascular obstruction and abnormal hemodynamics, it causes fixed pain in the pelvis. Thus, an optional therapy of SPID is to normalize the blood-stasis microcirculation in the inflammatory focus.

DZD, as a Chinese medicinal formula, is composed of* Salvia miltiorrhiza Bge., Morus alba L., Liguisticum chuanxiong Hort., Dipsacus asper Wall, Forsythia suspensa Vahl, Litchi chinensis Sonn., Corydalis turtschaninovii Bess. y. yanhusuo Y. H. Chou et C. C. Hsu, *and* Cyperus rotundus L.* as shown in Table 1. It is effective in dispersing blood stasis and dredging collaterals. In the previous study, we confirmed that DZD could decrease the level of VEGF-A/C/D expressions in endometrial cells in a 3D endometrial cell model. In this way, DZD could regulate endothelium function and improve the blood-stasis microenvironment in SPID [4].

In this study, we would observe MGS and blood flow of the pelvis (including ovarian artery, internal iliac artery, and mesenteric and pelvic wall microvessels) in the SPID murine model, to investigate whether DZD could relieve CPP and hemodynamics of the pelvis. Expressions of VEGF, Ang-2, and OPN were measured to evaluate DZD's influence in the anti-inflammatory process.

## 2. Materials and Methods

*Ethics Statement*. All animal experiments were carried out in strict accordance with the guidelines of the Institute of Chinese Materia Medica China Academy of Chinese Medical Sciences Animal Care and Use Committee (license number: SYXK (Jing) 2013-0035).

### 2.1. Animals

6–8-week-old female BALB/c mice were purchased from Vital River Laboratories and housed at the Institute of Chinese Materia Medica China Academy of Chinese Medical Sciences China institute of traditional Chinese medicine academy of sciences. The mice were kept under controlled temperature (23 ± 3°C) with 45%–65% humidity.

### 2.2. Preparation of DZD

The crude drugs of DZD were purchased from the pharmacy of Dongfang Hospital of Beijing University of Traditional Chinese Medicine. Soaked in 8 volumes of double-distilled water for 20 min, all the herbs in proportion were boiled and then decocted over a low heat for 20 min. Repeat twice; the decoction was homogenized and concentrated by heating until the concentration of the crude drug was 1.8 g/mL (high dose), 0.9 g/mL (mid dose), and 0.45 g/mL (low dose), respectively.

### 2.3. HPLC and Fingerprint Analysis

DZD of 0.9 g/mL was concentrated at 60°C in a rotary evaporator under reduced pressure. The yield of final extract was 25.07%. 0.2261 g of the sample was dissolved in 80% methanol, ultrasonicated, and then filtered on a 0.45 *μ*m filter for HPLC analysis. The extract of DZD was analyzed by Waters 2695 HPLC instrument (2998PDA detector). The sample was separated on a Waters Symmetry C18 column (4.6 × 250 mm, 5 *μ*m) and the linear gradient was 5–95% B (A = 0.1% formic acid water, B = acetonitrile) for 70 min. The flow rate was 1.0 mL/min and the column temperature was maintained at 30°C. Sample of 10 *μ*L was detected at 280 nm.

Reference compounds consisted of mulberroside A, phillyrin (purchased from Chengdu Pulse Biological Technology Co., Ltd.), tetrahydropalmatine, ferulic acid, and tanshinone IIA (purchased from National Institute for the Control of Pharmaceutical and Biological Products, Beijing, China). The method of HPLC was the same as above.

### 2.4. The Murine Model of SPID and Animal Treatment

Oestrus was identified by vaginal smear for continuous 3 cycles. Only those with regular cycles were allowed to continue the next experiments. Suitable mice were randomly allocated into 2 groups: a group of 30 mice served as the control group, and the others were used for the model of SPID.

Briefly, except the mice in the control group, the others were anesthetized with an intraperitoneal administration of 5% chloral hydrate at 3.5 mL/kg body weight and intrauterinely injected with* C. muridarum* Everette et al. (Nigg, ATCC VR-123) [7–9] of 10^5^ IFUs in 20 *μ*L SPG (sucrose-phosphate-glutamate buffer) to each uterine horn.* C. muridarum* was propagated, purified, and titrated as described elsewhere [10]. Aliquots of the organisms were stored at −80°C till use.

After 7 days, vaginal swabs were taken from 30 mice at random to carry on* C. muridarum* detection. Each swab was suspended in 500 mL of ice-cold SPG followed by vortexing with glass beads, and the released organisms were titrated on HeLa cell monolayers in duplicate until the titer showed negative. The number of IFUs/swab was converted into log_10_ and the log_10_ IFUs was used to calculate mean ± SEM [10].

Six weeks after infection, successfully infected 150 mice were randomly divided into 5 groups as follows: the model group, aspirin group, and high-dose, mid-dose, and low-dose DZD groups (DZD-H, DZD-M, and DZD-L, resp.). Each consisted of 30 mice.

All mice received drugs intragastrically at a dosage of 0.1 mL/10 g/day for 3 estrous cycles. The mice in the model group and the control group received 5‰ carboxymethylcellulose (CMC); in the aspirin group, aspirin 10 mg/(kg·d) was dissolved in 5‰ CMC; in the DZD-H, DZD-M, and DZD-L groups, DZD was dissolved at 18 g/(kg·d), 9 g/(kg·d), and 4.5 g/(kg·d), respectively.

### 2.5. Evaluation of CPP with MGS

Mice were captured and fixed with the routine method, and a Q-tip was used to press the bilateral lower quadrants of the abdomen in an appropriate intensity for 10 seconds. One researcher, who was blind to the grouping, operated the abdominal stimulation and scored mice's responses according to the MSG standard. The facial expressions included orbital tightening, nose bulge, cheek bulge, and whisker change. Based on different intensities, each feature was coded on a three-point scale that was 0 (not present), 1 (moderately visible), and 2 (severe) [11]. Another took charge of recording the grouping and the MGS score.

### 2.6. Assessment of Hemodynamics

By observation of vaginal smears, only mice in estuation were selected to assess the following tests in order to avoid the influence of hormone. All mice underwent the following tests in 5 days.

#### 2.6.1. Blood Flow of Ovarian and Internal Iliac Arteries

The blood flows of the ovarian and the internal iliac arteries were measured by the Visual Sonics Vevo 2100 high-resolution ultrasound microimaging system (Visual Sonics, Toronto, Canada) with a 40 MHz probe.

The mice were anesthetized with isoflurane vapor to minimize breathing movement during scans [12]. All hair was removed from the abdomen and lower back with a chemical hair remover [13]. Before imaging, coupling gel was applied between the skin and the probe.

In B-mode, the ovary was identified by locating the kidney firstly when the mouse was in the prone position; each ovary was behind its ipsilateral kidney [12]. The ovarian artery was selected to test blood flow in color mode, and peak systolic velocity (PSV) was measured to be compared between groups.

The internal iliac artery was found when the mouse was in the supine position, which was below the bladder and traced by locating the abdominal aorta and unilateral common iliac artery firstly. In the same way, blood flow and PSV of the internal iliac artery were assessed.

#### 2.6.2. Microvascular Perfusion of Pelvic Wall

Blood flow of pelvic wall was invasively assessed with the flowmeter PeriFlux 5000 (PeriCam PSI, Sweden), utilizing the Doppler shift, that is, the frequency change that laser light undergoes when interacting with objects in motion [14]. Three points of microvessels of the pelvis were selected randomly to test blood perfusion; the average was taken to evaluate the local microcirculation.

#### 2.6.3. Mesenteric Blood Flow Perfusion

This study was carried on with the PeriCam PSI system. It is a blood perfusion imager based on the Laser Speckle Contrast Analysis (LASCA) technology [15]. In the pelvis, intestines of similar length were chosen to conduct the PSI test. A piece of black paper was placed under the intestines to avoid irrelevant blood flow signals: sample rate of 1 frame per second and detecting distance of 10.5 ± 0.5 cm with the whole monitoring area of 3 × 3 cm^2^. Perfusion data was recorded in real time, and the date of the Region of Interest (ROI) was calculated and analyzed by the PSI system [15].

### 2.7. Western Blot Analysis

Tissue samples of the upper genital tract were homogenized and lysed in ice-cold RIPA lysis buffer (C1053+; Applygen Technologies, Beijing, China) supplemented with protease inhibitor (P1265; Applygen Technologies). The proteins were in the supernatants after the homogenates were centrifuged at 12000 ×g for 20 min at 4°C. The proteins were quantified with bicinchoninic acid (BCA) (P1511; Applygen Technologies) and resolved in SDS-PAGE loading buffer at 95°C for 10 min. After loading (20 *μ*g protein), proteins were separated in 10% Tris-Bis gel and then transferred to nitrocellulose membranes by electroblotting. After blotting in 5% of nonfat dry milk in TBST for 30 min at room temperature, the membranes were incubated with anti-VEGF (1 : 1000, ab46154; Abcam, Cambridge, UK) or anti-Ang-2 (1 : 1000, SC-20718; Santa Cruz Biotechnology, Heidelberg, Germany) or anti-OPN (1 : 1000, ab8448; Abcam, Cambridge, UK) antibodies overnight at 4°C, followed by incubation with goat anti-rabbit horseradish peroxidase-labeled secondary antibody (1 : 5000, C1309; Applygen Technologies) for 1 hour at room temperature. The blots were visualized using the Super ECL Plus detection reagent (P1010; Applygen Technologies). The enhanced chemiluminescence signals were detected using Quantity One software (Bio Rad).

### 2.8. Immunohistochemistry

The upper genital tract tissues were immersed in 4% paraformaldehyde. Formalin-fixed tissues were separately paraffin-embedded and cut into 5 *μ*m sections, stained with anti-VEGF (1 : 100) or anti-Ang-2 (1 : 100) or anti-OPN (1 : 200) as mentioned above. DAB was used as chromogen. Negative controls were run by omitting the primary antibodies. Images were photographed with digital camera (E4500; Nikon, Mississauga, Ontario, Canada) at ×40 magnification on a light microscope (Nikon Eclipse E600). The mean IOD values of VEGF, Ang-2, and OPN were calculated with Image-Pro Plus 6.0.

### 2.9. Statistical Analyses

Data was presented as the mean ± SEM. Statistical analysis was performed using SPSS 19.0 and one-way ANOVA was used to compare the data between the 6 groups. Correlation coefficients were calculated to identify the relationship between CPP and hemodynamics of the pelvis. Difference was considered significant at *P* < 0.05.

## 3. Result

### 3.1. Identification of Compounds

**Figure 1:**
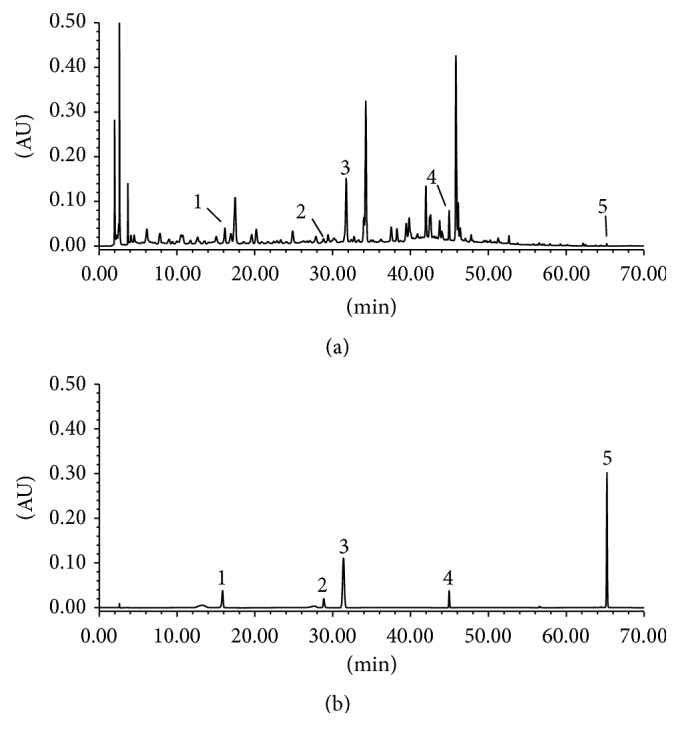
The HPLC-based fingerprint of DZD. (a) DZD of 0.9 g/mL; (b) mixed reference compounds. 1: mulberroside A; 2: tetrahydropalmatine; 3: ferulic acid; 4: phillyrin; 5: tanshinone IIA.

As shown in Figure 1, these compounds of DZD were identified as mulberroside A; tetrahydropalmatine; ferulic acid; phillyrin; tanshinone IIA. The established method could identify the bioactive compounds in DZD at the same time.

### 3.2. Verification of Infection

7 days after infection, vaginal swabs were collected from 30 mice randomly. The log_10_ IFUs/swab was significantly different with the data before infection (4.70 ± 0.64 versus 0). It verified that mice were successfully infected with* C. muridarum* intrauterinely.

### 3.3. Treatment with DZD Relieved CPP in Mice with SPID

The result was shown in Table 2. Based on the data, it could be stated that CPP formed in the infected mice, as the MGS score was significantly different between the control group and the model group. High-dose and mid-dose DZD could obviously relieve CPP and were superior to aspirin. Low-dose DZD had similar effect with aspirin, which could not completely relieve CPP in SPID mice.

### 3.4. Treatment with DZD Accelerated Hemodynamics in Mice with SPID

**Figure 2:**
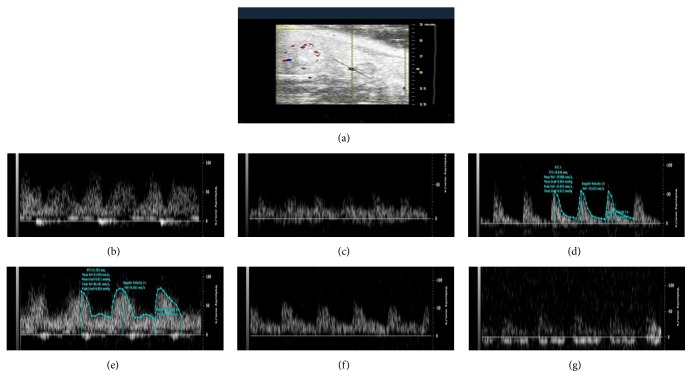
Representative Doppler waveforms of the ovarian artery. (a) The image of the ovary; (b) the control group; (c) the model group; (d) the aspirin group; (e) the DZD-H group; (f) the DZD-M group; (g) the DZD-L group.

**Figure 3 fig3:**
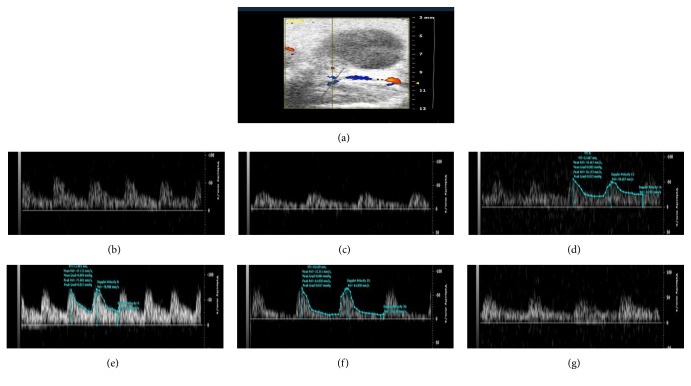
Representative Doppler waveforms of the internal iliac artery. (a) The image of measurement of the internal iliac artery in color mode; (b) the control group; (c) the model group; (d) the aspirin group; (e) the DZD-H group; (f) the DZD-M group; (g) the DZD-L group.

**Figure 4 fig4:**
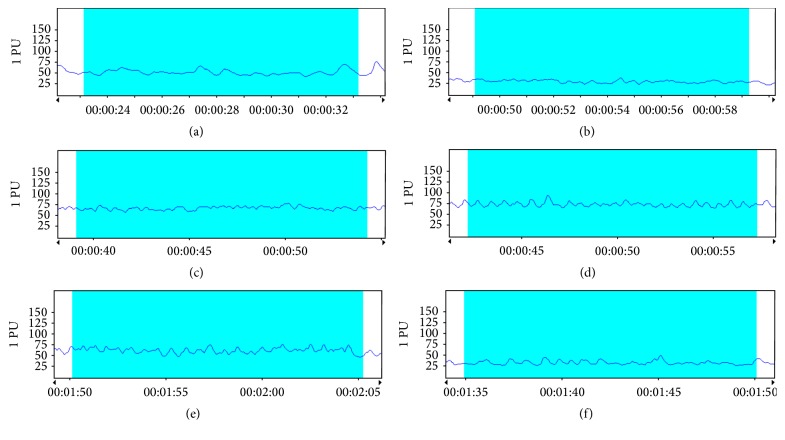
Representative blood perfusion curves of the pelvic microvessels. (a) The control group; (b) the model group; (c) the aspirin group; (d) the DZD-H group; (e) the DZD-M group; (f) the DZD-L group.

**Figure 5:**
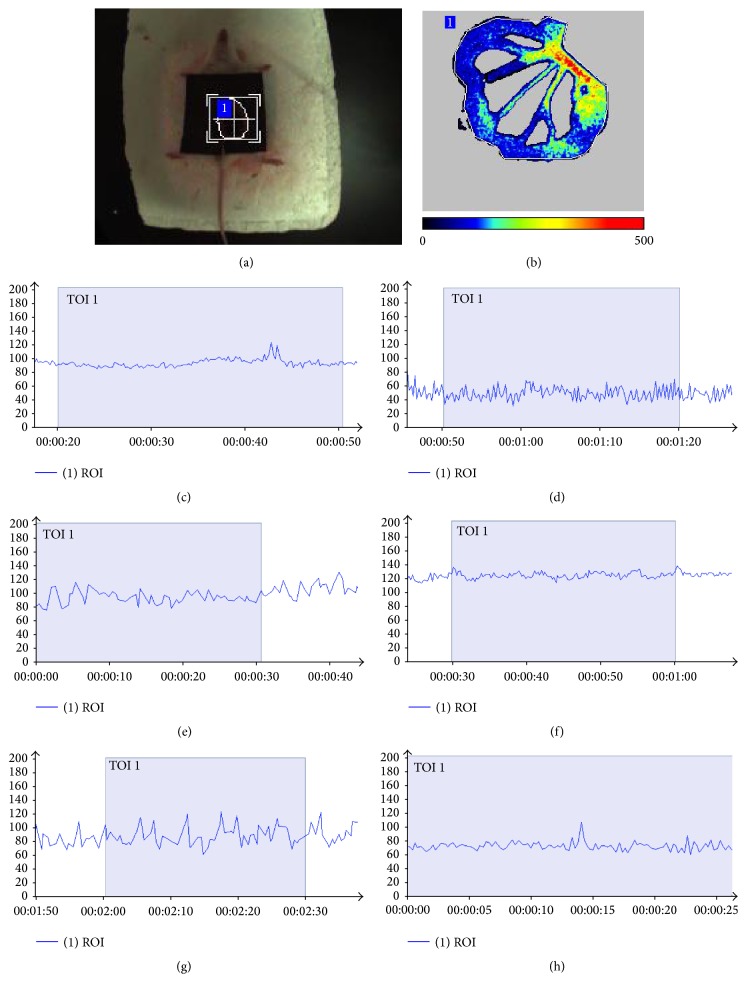
Representative blood perfusion curves of the mesentery. (a) The image of measurement of a mouse; (b) the image of Doppler signals of blood flow; (c) the control group; (d) the model group; (e) the aspirin group; (f) the DZD-H group; (g) the DZD-M group; (h) the DZD-L group.

The result was shown in Table 2 and Figures 2 [Fig fig3] [Fig fig4]–5. Blood flows, involving PSV of the ovarian artery and the internal iliac artery, blood flow perfusion of the pelvic wall, and mesentery in the pelvis, were significantly different between the mice in the model group and the control group. It could be concluded that the pelvis was in blood-stasis microenvironment in SPID mice. Treatment with high-dose and mid-dose DZD accelerated blood flow in the pelvis; the blood-stasis environment could be improved significantly by dose-dependent DZD compared with the placebo (CMC). High-dose DZD increased blood flow significantly, even in excess of the level of the control group, which might cause other problems; mid-dose DZD had a similar positive effect with aspirin on blood circulation, while low-dose DZD could not significantly increase blood flow in the pelvis. So in SPID mice, mid-dose DZD could properly improve hemodynamics of the pelvic blood-stasis microenvironment.

### 3.5. Correlation between MGS and Hemodynamics of the Pelvis in the SPID Murine Model

The MGS score statistically showed a significant negative correlation with PSV of the ovarian artery and the internal iliac artery, blood perfusion of microvessels of pelvic wall, and mesentery (*r* = −0.311, *P* = 0.000; *r* = −0.166, *P* = 0.026; *r* = −0.349, *P* = 0.000; *r* = −0.363, *P* = 0.000, resp.).

### 3.6. Treatment with DZD Decreased Ang-2, VEGF, and OPN Levels in the Upper Genital Tract

The results were evaluated by Western blot and immunochemistry.

#### 3.6.1. Western Blot Analysis Results

**Figure 6:**
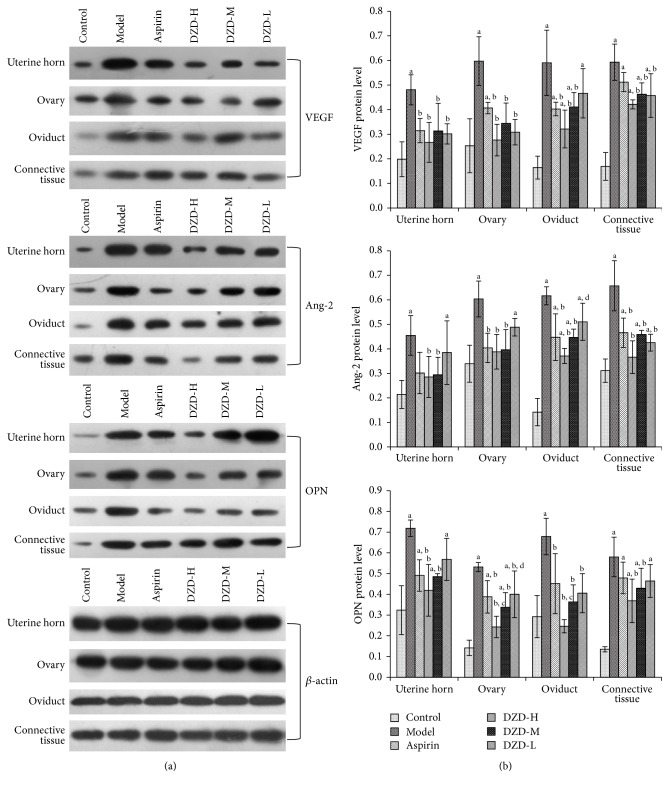
Protein expressions in the upper genital tract. Western blot was used to measure VEGF, Ang-2, and OPN protein levels in the upper genital tract of different groups (*n* = 5). ^a^ *P* < 0.05 compared with the control group; ^b^ *P* < 0.05 compared with the model group; ^c^ *P* < 0.05 compared with the aspirin group; ^d^ *P* < 0.05 compared with the DZD-H group.

The protein expressions of VEGF, Ang-2, and OPN in the upper genital tract were confirmed by Western blot (Figure 6). The protein levels were normalized by *β*-actin. Among the 6 groups, VEGF, Ang-2, and OPN levels in the model groups were significantly higher than those in the control groups in the upper genital tract (including the uterine horn, ovary, oviduct, and connective tissue). These protein levels decreased in the aspirin, DZD-H, DZD-M, and DZD-L groups compared with those in the model groups, but significant differences were more common in the DZD-H and DZD-M groups. There were no significant differences between the DZD-H and DZD-M groups.

#### 3.6.2. Immunochemical Analysis Results

To verify the changes of the protein levels, the mean IOD values of VEGF, Ang-2, and OPN expressions were measured using immunochemistry. The results were shown in Table 3. The mean IOD of VEGF, Ang-2, and OPN in the model groups was significantly increased compared with those in the control groups. The mean IOD of these protein expressions was lower in DZD and aspirin groups compared with the model groups, but more significant differences were found in the DZD-H and DZD-M groups. Significant intergroup differences were not seen in the 2 groups.

### 3.7. Gastrointestinal Adverse Effects

During the study, mice were not observed any gastrointestinal symptoms in all groups (including dyspepsia, gastrointestinal bleeding, etc.). After being sacrificed, the digestive tract of mice was checked and none of gastrointestinal mucosal defects were detected either.

## 4. Discussions

SPID is increased by delayed antimicrobial treatment of PID [16] and its occurrence cannot be excluded by short-term clinical and/or microbiologic cure [17]. Its manifestations include infertility, ectopic pregnancy, and chronic pelvic pain as described before and can cause considerable physical and emotional problem, in addition to significant cost on healthcare services [18].

For SPID, early prevention is essential, mainly involving health-care education that emphasizes the importance of safe sex and encourages early presentation for the diagnosis [19] and timely and normative use of broad-spectrum antibiotics in accordance with current management guidelines in acute episode. Once SPID forms, nonsteroid anti-inflammatory drugs (NSAIDs) play a limited role in the treatment because of pelvic adhesion and fibrosis [20]. Also, a long-time, repeating, and excessive use of NSAIDs in SPID has obvious adverse effects in the body, mainly involving gastrointestinal injury, such as dyspepsia, ulcer, and gastrointestinal bleeding. Besides these, renal impairment, interactions with other commonly prescribed medications, weakened immunity, and drug resistance could be observed [21, 22]. Traditional Chinese formula, acupuncture and moxibustion, short wave diathermy, acupoint injection, retention enema, and so forth are effective on the treatment of SPID as reported in lots of researches. Among these therapies, traditional Chinese formula for oral administration is relatively more commonly used in China.

As mentioned above, blood stasis is considered the main pathogenesis of SPID. It is caused by interruption of qi and blood in the pelvis because of invasion and retention of external or internal pathogenic factors in the pelvic collaterals [23]. At the meantime, blood stasis exacerbates stagnant movement of qi and blood. As a pathogenic factor, it causes persistent pelvic pain and other dysfunctions of the genital tract [24]. Therefore, the principle of SPID treatment is to disperse blood stasis and dredge collaterals.

DZD was formulated in accordance with the principle and in combination of herbs with doctors' individual experience.* Salvia miltiorrhiza Bge*. and* Morus alba L*. are main components of DZD. Both of them have antibacterial effect [25, 26] in modern pharmacological studies. The former also plays a role in expansion of arteries and improvement of microcirculation. The other herbs assist in anti-inflammatory effect or increasing blood flow in vessels or alleviating pain [27–32]. Combination of herbs is thought to increase therapeutic efficacy and reduce adverse effects through multiple targets and biological pathway [33], which can be confirmed by identification of bioactive compounds in the formula. As a result, waste products, such as prostaglandin [34], are removed from the blood circulation; pain threshold is lifted; nutrition and oxygen supply is increased; metabolism is enhanced to facilitate absorption and elimination of inflammation; tissue fibrosis is softened. Thus series of symptoms can be relieved.

CPP is a common symptom of SPID. It occurred in about 36% PID patients in the PEACH trial [35]. In clinical trials, pain could be graded by Visual Analogue Scale (VAS) [36] or Chronic Pain Grade developed by Von Korff et al. [37], both of which were based on patients' language expression. In animal experiments, the severity of pain cannot be expressed, so normal methods are unsuitable to be applied [38]. In the study, MGS, standardized behavioral coding system involving noxious stimuli of moderate duration accompanied by facial expressions of pain [11], was used to assess CPP in mice. The results of MGS in the control, high-dose, and mid-dose DZD groups had no significant difference between each other and were significantly lower than those of the low-dose DZD and the aspirin groups. It suggested that certain dose of DZD could dramatically relieve CPP in SPID mice.

Hemodynamics is an important parameter in many conditions of gynecology and obstetrics, so as to predict the risk of pregnancy [39], judge the severity of adnexal torsion [40], assess the function of pelvic floor [41], and so forth. In this study, we analyzed (1) peak systolic velocity (PSV) of the ovarian artery and the internal iliac artery and (2) blood perfusion of microvessels of pelvic wall and mesentery, to observe the hemodynamics of SPID and to evaluate the effect of DZD on pelvic blood flow. According to the present results, especially the results of the model group, we concluded that, in SPID, pelvis was in the blood-stasis microenvironment, which is consistent with TCM theory as mentioned above. By eliminating blood stasis and promoting blood circulation, DZD could improve hemodynamics and blood-stasis microenvironment in the pelvis of SPID when it was in a suitable dose (mid-dose).

The results also indicated a negative correlation between CPP and hemodynamics of the pelvis in SPID mice. It was confirmed that CPP was related to obstructed blood circulation. Eliminating blood stasis and promoting blood circulation are a selectable option for the treatment of SPID, especially the symptom, CPP.

VEGF, Ang-2, and OPN are well-known proinflammatory factors, distributed in a variety of tissues, including the reproductive tracts [42–44]. These factors are increased during inflammation, and the expression levels are positively correlated with the severity of inflammatory responses [45]. In this study, VEGF, Ang-2, and OPN expressions were significantly higher in the model groups. After the treatment of drugs, these factors declined to varying degrees, especially in the DZD-H and DZD-M groups, and all of the differences are statistically significant compared with the model groups. It was indicated that DZD might play a positive role in the anti-inflammatory process.

Aspirin is the positive control drug in this study, as it is a well-known NSAID, also widely used for its analgesic and platelet antiaggregation properties [46] to relieve pain and improve blood circulation. We observed that aspirin had similar effect with mid-dose DZD on the aspect of hemodynamics in the pelvis, but it was less effective than mid-dose DZD in terms of alleviating CPP and downregulating proinflammatory factors.

Our study explained the following results: (1) DZD could relieve CPP in SPID; (2) the pelvis was in blood-stasis microenvironment in SPID mice; (3) DZD could improve the blood-stasis microenvironment by accelerating hemodynamics in the pelvis, including arteries of upper genital tract, microvessels in the pelvic wall, and mesentery; (4) CPP had correlation with obstructed blood flow in some ways and promoting blood circulation could relieve CPP; (5) DZD could reduce the expressions of VEGF, Ang-2, and OPN in the upper genital tract.

In conclusion, dose-dependent DZD relieved CPP and improved the blood-stasis microenvironment of SPID by ameliorating the hemodynamics in the pelvis. Moreover, it might play a positive role in the anti-inflammatory process by downregulating proinflammatory factors. A further clinical evaluation needs to be conducted to confirm these results in human subjects.

Additionally, in clinical setting, PID patients are firstly prescribed with sensitive antibiotics in the acute phase. However, in the chronic stage, antibiotics' effect is limited. Furthermore, aspirin is more effective on blood flow. So the study did not set a control group of antibiotics. This may be limitation to completely mimic the real world. Gastrointestinal adverse effects of both DZD and aspirin have not been found in this animal experiment. In clinical practice, patients should be monitored mindfully to ensure medication safety.

## Figures and Tables

**Table 1:** The composition of Danzhi decoction (DZD).

Scientific name	Chinese name	Medicinal parts	Grams	%
*Salvia miltiorrhiza Bge.*	Danshen	Root	10	13.0

*Morus alba L.*	Sangzhi	Twig	10	13.0

*Liguisticum chuanxiong Hort.*	Chuanxiong	Tuber	6	7.8

*Dipsacus asper Wall*	Chuanduan	Root	15	19.5

*Forsythia suspensa Vahl*	Lianqiao	Fruit	10	13.0

*Litchi chinensis Sonn.*	Lizhihe	Seed	10	13.0

*Corydalis turtschaninovii * *Bess. y. yanhusuo Y. H.* *Chou et C. C. Hsu*	yuanhu	Root	10	13.0

*Cyperus rotundus L.*	Xiangfu	Tuber	6	7.8

Total amount			77	100

**Table 2:** MGS score and hemodynamic parameters in each group.

	Control group	Model group	Aspirin group	DZD-H group	DZD-M group	DZD-L group
MGS	1.367 ± 1.245	5.633 ± 1.847^a^	3.167 ± 1.315^a,b^	2.033 ± 1.098^b,c^	1.667 ± 1.093^b,c^	2.900 ± 1.242^a,b,d,e^
Blood flow of internal genital tract						
PSV of ovarian artery	58.288 ± 11.853	45.772 ± 10.880^a^	55.219 ± 15.668^b^	60.400 ± 16.312^b^	58.326 ± 9.813^b^	46.538 ± 6.393^a,c,d,e^
PSV of internal iliac artery	51.176 ± 7.778	44.685 ± 9.038^a^	51.516 ± 12.975^b^	59.286 ± 14.137^a,b,c^	51.431 ± 10.211^b,d^	44.069 ± 8.650^a,c,d,e^
Microvascular perfusion of pelvic wall	53.783 ± 7.454	31.596 ± 7.682^a^	60.992 ± 11.219^b^	70.380 ± 15.757^a,b,c^	56.901 ± 9.801^b,d^	37.735 ± 6.219^a,c,d,e^
Mesenteric blood flow perfusion	90.596 ± 13.594	61.029 ± 21.219^a^	91.209 ± 22.603^b^	107.375 ± 22.902^a,b,c^	85.818 ± 17.079^b,d^	58.926 ± 15.59^a,c,d,e^

^a^ *P* < 0.05 compared with the control group; ^b^ *P* < 0.05 compared with the model group; ^c^ *P* < 0.05 compared with the aspirin group; ^d^ *P* < 0.05 compared with the DZD-H group; ^e^ *P* < 0.05 compared with the DZD-M group.

**Table 3:** The mean IOD in each group.

	Control group	Model group	Aspirin group	DZD-H group	DZD-M group	DZD-L group
VEGF						
Uterine horn	54.505 ± 16.039	135.296 ± 29.325^a^	51.799 ± 12.086^b^	51.742 ± 15.186^b^	54.220 ± 14.616^b^	57.915 ± 16.543^b^
Ovary	59.378 ± 17.142	144.959 ± 37.763^a^	96.791 ± 15.833^a,b^	57.886 ± 15.867^b,c^	59.343 ± 16.402^b,c^	69.018 ± 20.919^b^
Oviduct	94.771 ± 41.003	529.083 ± 236.206^a^	157.738 ± 32.198^b^	145.077 ± 45.462^b^	150.045 ± 45.070^b^	462.198 ± 173.323^a,c,d,e^
Connective tissue	73.784 ± 14.413	602 ± 160.033^a^	563.536 ± 198.160^a^	244.358 ± 123.487^b,c^	207.859 ± 75.754^b,c^	216.703 ± 132.329^b,c^
Ang-2						
Uterine horn	23.019 ± 7.897	45.572 ± 3.830^a^	35.053 ± 12.983	26.587 ± 7.638^b^	27.498 ± 11.256^b^	40.213 ± 10.506^a,d,e^
Ovary	20.383 ± 4.929	97.899 ± 59.154^a^	27.711 ± 11.785^b^	21.635 ± 6.463^b^	31.504 ± 10.090^b^	79.445 ± 30.283^a,c,d,e^
Oviduct	27.482 ± 11.808	1443.231 ± 300.789^a^	364.597 ± 65.823^a,b^	330.218 ± 89.037^a,b^	416.294 ± 170.433^a,b^	1261.075 ± 244.432^a,c,d,e^
Connective tissue	65.812 ± 15.765	210.132 ± 56.290^a^	132.163 ± 52.801^a,b^	84.020 ± 13.940^b^	114.405 ± 36.019^b^	114.298 ± 41.990^b^
OPN						
Uterine horn	27.591 ± 5.723	81.30 ± 32.775^a^	54.024 ± 5.950^a,b^	33.113 ± 5.556^b^	52.330 ± 3.519^b^	81.801 ± 31.474^a,c,d,e^
Ovary	142.331 ± 39.000	416.631 ± 84.065^a^	228.653 ± 67.869^b^	162.881 ± 19.448^b^	225.215 ± 75.143^b^	302.368 ± 122.731^a,b,d^
Oviduct	198.387 ± 127.226	532.347 ± 172.325^a^	195.368 ± 86.126^b^	166.968 ± 58.035^b^	206.683 ± 74.969^b^	205.345 ± 67.066^b^
Connective tissue	110.077 ± 48.041	482.171 ± 135.153^a^	415.074 ± 84.211^a^	241.861 ± 103.486^a,b,c^	218.142 ± 65.258^b,c^	434.350 ± 126.390^a,d,e^

^a^ *P* < 0.05 compared with the control group; ^b^ *P* < 0.05 compared with the model group; ^c^ *P* < 0.05 compared with the aspirin group; ^d^ *P* < 0.05 compared with the DZD-H group; ^e^ *P* < 0.05 compared with the DZD-M group.
